# Outcome of multifaceted interventions for improving the quality of antenatal care in Nigerian referral hospitals

**DOI:** 10.1186/s12978-020-00997-6

**Published:** 2020-11-04

**Authors:** Friday Ebhodaghe Okonofua, Lorretta Favour Chizomam Ntoimo, Bola Ekezue, Victor Ohenhen, Kingsley Agholor, Mohammed Gana, Brian Igboin, Chioma Ekwo, Wilson Imongan, Hadiza Galadanci, Rosemary Ogu

**Affiliations:** 1 Women’s Health and Action Research Centre (WHARC), Benin City, Nigeria; 2 Centre of Excellence in Reproductive Health Innovation, University of Benin, Benin City, Nigeria; 3 Department of Obstetrics and Gynaecology, University of Benin and University of Benin Teaching Hospital, Benin City, Nigeria; 4 Federal University, Oye, Ekiti Nigeria; 5 Fayetteville State University, Fayetteville, USA; 6 Central Hospital Benin City, Benin City, Nigeria; 7 Central Hospital, Warri, Delta State Nigeria; 8 General Hospital Minna, Minna, Niger State Nigeria; 9 Bayero University, Kano, Nigeria; 10 University of Port Harcourt, Port Harcourt, Rivers State Nigeria

**Keywords:** Antenatal care, Maternal mortality, Nigeria, Quality of care, Referral hospitals

## Abstract

**Background:**

The study was designed as quasi-experimental research to investigate the effectiveness of multifaceted interventions for improving the quality of antenatal care in referral hospitals in Nigeria. Two referral hospitals (the Central Hospital in Benin City, South-South Nigeria, and the General Hospital in Minna) served as intervention sites, while two hospitals in comparable locations, (the Central Hospital Warri and the Suleja Hospital Abuja) were the control hospitals.

**Methods:**

Intervention activities consisted of the introduction of a strategic plan with the shared vision of reducing maternal mortality by 50% in 1 year in the hospitals; staff training and re-training; the establishment of an automated appointment system; composite health education involving couples and providers; advocacy with policymakers; and the implementation of maternal death reviews and surveillance. These activities were implemented in the intervention hospitals over 21 months (October 2017 to June 2019). Exit interviews of pregnant women at intervention and control sites by trained interviewers were used to assess the quality of antenatal care after their visit, A total of 777 women were interviewed (427 in the intervention sites and 350 in the control sites). Data were analyzed with univariate and multivariate Poisson and logistic regression to determine the extent to which health providers in the clinics completed the 18 signal functions identified in the WHO assessment tool.

**Results:**

The regression analyses showed the interventions were effective in improving six quality indicators (QIs) for counseling and information sharing. The difference between intervention and control sites on these QIs was significant at < 0.05. On the contrary, the interventions were less effective for maternal and fetal measurements; and disease testing and management QIs.

**Conclusion:**

The positive effects of the interventions are likely due to the effectiveness of the training and health education components. The lack of intervention impact observed for maternal and fetal measurements may be due to the high workload of care staff and inadequate clinic supplies, which we did not address. We conclude that interventions that address the quality of antenatal care in low-resource settings should focus on improving all elements of care, including adequate staffing and mobilization of material resources.

**Trial registration:**

This study was registered in the ISRCTN on August 14th, 2020. Trial Registration Number.

SRCTN17985403. Retrospective registration. The reason for the retrospective registration is the current non-recognition of the Nigeria Clinical Trials Registry (NCTR); which is currently not an ICMJE or WHO ICTRP approved registry. (This study was registered in the Nigeria Clinical Trials Registry on April 14th, 2016. Trial Registration Number NCTR No: 91540209).

## Plain English summary

The World Health Organization (WHO) recommends antenatal care for pregnant women to ensure the early detection and treatment of complications that result in adverse birth outcomes. We had evidence from formative research that poor quality antenatal care may contribute to the high rate of maternal deaths, especially in booked patients in Nigeria. We, therefore, designed implementation research to determine the effectiveness of a set of multifaceted interventions in improving the quality of antenatal care in referral hospitals. Two hospitals – Central Hospital, Benin and the General Hospital, Minna – were the intervention sites where the interventions were applied, while two hospitals in contiguous areas – the Central Hospital Warri, and the Suleija Hospital, Abuja – were the control hospitals where interventions were not applied. The interventions included a strategic plan with the shared vision of reducing maternal mortality by 50% in 1 year in the hospitals, staff re-training, the establishment of an automated appointment system; fused health education involving couples and providers; advocacy with policymakers; and maternal death reviews and surveillance. After 21 months, we compared the results in terms of the propensity for staff in the hospitals to carry out 20 WHO recommended antenatal signal functions. The results showed that the interventions were effective in improving the quality of counseling and information sharing but were less effective in maternal and fetal measurements and disease testing and management. We conclude that interventions to improve the quality of antenatal care would be more effective if the supply side of service delivery is also improved.

## Background

With an estimated 60,000 annual maternal deaths and a maternal mortality ratio of 814 per 100,000 births, Nigeria is currently ranked by the World Health Organization as one of the countries with the highest rates of maternal mortality globally [1, 2]. Along with India, Nigeria currently accounts for one-third of annual maternal deaths in the world [2]. Among several other factors, the inadequate use of skilled maternity care providers by pregnant women has featured prominently as a major determinant of the high rate of maternal deaths in Nigeria [3–5]. Data from the 2018 Nigerian Demographic and Health Survey (NDHS) indicate that only 67% of Nigerian women received at least one antenatal care from a skilled provider during their pregnancy, while a significant proportion received no care. Several published reports from the early 1980s to date indicate that a large proportion of maternal deaths in the country occur in women who did not receive antenatal care in orthodox clinical settings [5–10]. Consequently, considerable resources and priority have been devoted over the past years to increase ‘women’s access to skilled pregnancy care in primary health care settings or referral hospitals [11–13].

However, increasingly worrisome is the equally high number of women during pregnancy who die despite receiving antenatal care in orthodox clinics and hospitals [5, 8, 10]. A recent study from a maternal and perinatal death review and surveillance in southwest Nigeria, [14] reported that up to 50% of recorded maternal deaths occurred in women who received antenatal care in health facilities. We posit that the high rate of maternal deaths in women receiving antenatal care is most likely attributable to poor quality antenatal care offered in those settings, or to inadequate compliance of pregnant women to services offered in antenatal clinics. Poor adherence of women to prescribed antenatal services may also be due to the quality of counseling and staff-client interactions in the clinics. It is therefore critical to identify ways to improve the quality of antenatal care as a critical strategy for reducing the high rate of maternal mortality in the country.

To date, the quality of antenatal care offered within ‘Nigeria’s health care system has not been systematically investigated. A recent paper based on an analysis of the 2013 Nigeria DHS showed that several women who reported having used antenatal care in the survey did not receive the full complement of services recommended by the Federal Ministry of Health of Nigeria as well as the guidelines recommended by the WHO for quality antenatal care [15]. Indeed, the authors recommended the improvement of the quality of antenatal care services as one of the key strategies for reducing the high rate of maternal mortality in the country.

Despite the adoption of primary health care as the entry point into ‘Nigeria’s health care system by the Federal Ministry of Health, up to 70% of the antenatal care services in Nigeria are delivered by referral facilities – secondary and tertiary hospitals. In 2015, we began a comprehensive intervention program aimed at improving the quality of skilled pregnancy care in ‘Nigeria’s referral hospitals with the overall goal to reduce maternal mortality. Our initial formative research in eight referral hospitals in four geo-political zones of the country identified several factors that were perceived by women and health providers as associated with poor quality antenatal, delivery and postnatal care in the hospitals. These included 1) the lack of official commitment to quality care [16];2) limited ‘providers’ skills and knowledge [17]; 3) long waiting hours in the antenatal clinics [18]; 4) abusive care by providers and poor ancillary services [19], and 5) heavy provider workloads [20].

To address these challenges, we designed and have implemented a multiple set of interventions since October 2017 in partnership with various stakeholders, including policymakers, hospital administrators, health providers, clinicians, and pregnant women and their spouses. The intervention focused on improving the delivery of content and experience of care according to the WHO framework for quality antenatal care [21, 22], among others. Prospective data collection occurred at intervention and control hospitals over 21 months. The objective of this paper is to report the results of the analysis comparing the indicators of quality of antenatal care between two intervention hospitals with two control hospitals of similar status where the interventions were not carried out. We believe the results of this study will be useful for developing substantive policies and practices for improving the quality of antenatal care in Nigeria.

## Study design and methodology

The study was a quasi-experimental research design whereby specific interventions were implemented in two referral hospitals (one in southern Nigerian and the other in northern Nigeria), while two referral hospitals in comparable locations in southern and northern parts of the country served as the control hospitals.

### Intervention vs. control hospitals

The Central Hospital in Benin City, South-South Nigeria, and the General Hospital in Minna, Niger State, in the North-central part, served as the intervention hospitals. By contrast, the Central Hospital Warri, South-South Nigeria, and the Suleja General Hospital, Abuja, in the North-Central region served as the control hospitals. The four hospitals are large referral hospitals that serve large populations of women in four States and two geo-political zones of Nigeria. We decided to use two intervention and two control hospitals for ease of management, to ensure data accuracy and to maximize local efforts. The Central hospitals in Benin City and Warri are only 80 km apart and serve similar populations of women. As such, we assumed there are no substantial population differences that will jeopardize the comparability of the data between the two hospitals. Similarly, the Suleja and Minna hospitals are in the same sociocultural and geographical area and are only 120 km apart, which suggests that any differences may not be cultural.

### Intervention activities

Beginning from October 2017, we started the implementation of a series of interventions to be deeply embedded in the sustainable workings of the two intervention hospitals in Benin City and Minna. These included:

1. The development of a strategic plan document with the hospital managers, health providers, and policymakers responsible for policy oversight. This activity took place over 3 months before the commencement of the intervention in October 2017. A strategic document was shared with all staff and health providers, with the goal of reducing maternal mortality by 50% in the two hospitals over 2 years. We discussed the challenges associated with providing optimal maternal care in the hospitals and identified shared strategies in the strategic plan. A workshop was delivered in the intervention hospitals to disseminate the strategic plan to the staff.
2. Staff training and re-training: We developed a three-day workshop to provide knowledge and skills training for doctors and midwives. The training focused on the provision of maternal health care, including antenatal, delivery, and postnatal care. Attendees of the workshop received training on respectful and non-abuse care, patient counseling, the use of treatment algorithms for decision-making, risks assessment and management, and the management of specific complications of pregnancy. A multimodal approach that includes lectures, discussions, role-plays, demonstrations, and hands-on sessions was used.
3. Computerized appointment system: To reduce the time spent during visits to the hospital by women for antenatal care, we developed and implemented an automatic appointment system. It involved computerization of all the records of the women. Each registered woman was prescheduled for visits to the clinic for antenatal care. The woman received automatic multiple reminders before their scheduled date of antenatal visit. This is to reduce missed visits and to improve pre-visit planning by the doctor and reduce wait times. Also, the appointment system will help in managing the ‘doctors’ workload and allow them to provide quality care.
4. Implementation of a composite health education program for the women and their spouses: As part of the intervention, we redesigned the health education for pregnant women to be delivered outside antenatal clinic hours in order to reduce the time often wasted on providing health education to women on clinic days. We introduced a monthly health talk program on Saturdays over 3 h that allowed adequate time to provide relevant information and also provide answers to questions raised by the women. Experts in the field provided the health talk and invited attendees were all pregnant women registered in the hospital at the time, their spouses, all health providers in the hospital, hospital managers, policymakers, and other interested persons. Program activities included the distribution of information leaflets and other Behavioral Change Communication (BCC) materials. We also developed a specific information booklet titled Answers to frequently asked questions by pregnant women. The booklet was developed from the questions the women raised during the first few months of the program. The booklet was printed and shared with the women and their spouses during the sections. The booklet was translated to the Hausa language and distributed to women in Niger State, who predominantly speak Hausa. We conducted 22 monthly health talks in the hospitals, with over 2500 pregnant women attending in total.
5. Maternal death reviews and surveillance: Clinical and nursing staff were trained to use the Federal Ministry of Health protocol for conducting maternal death reviews and surveillance (MDRS). The methodology has been reported elsewhere [23]. All maternal deaths that occurred in the hospitals during the period were reviewed to determine the medical and social causes of death. Thereafter, specific remedial measures were undertaken to correct the deficiencies in clinical management that led to maternal deaths.
6. Advocacy to policymakers and hospital administrators: We conducted advocacy visits to policymakers and health administrators to engender the need for resource allocation and disbursement for the provision of maternal health care.

### Data collection

We collected data prospectively on antenatal care utilization and quality of antenatal care from the two intervention hospitals and two control hospitals over 21 months period. All the women who presented for antenatal care within the period were eligible for inclusion in the study. During the formative phase of the project in 2015, a record of antenatal care for attendees was obtained. Fairly consistent records were available only for 3 years (2011–2013). The average number of attendees per month for the intervention and control hospitals was 5622. With this average, we estimated that about 118,062 women are likely to use the intervention hospitals as well as the control facilities for antenatal care in the next 21 months. Using ‘the Yamane’s formula [24] as shown below, a sample size of 800 was derived (400 for the experiment and 400 for the control hospitals).

$$ n=\frac{N}{1+N\left({e}^2\right)\kern0.75em } $$

where, N is the population size.

and e is the level of precision (±5%).

Assuming 95% confidence level and *p* = 0.5, and an estimated population of antenatal care attendees of 118,062 for each arm, we get the sample size as:

$$ n=\frac{\mathrm{118,062}}{1+\mathrm{118,062}\left({0.05}^2\right)\kern0.75em } $$ = 399.99

From the overall sample, we randomly selected ten women per month in both intervention and control hospitals. The exit interviews were conducted immediately after the women left the antenatal clinic locations, and by an interviewer who was not part of the clinical team that managed the patient. Consent was individually obtained from the women to conduct the interview after the details were fully explained to them. Only those who agreed to participate in the study were interviewed. In all, 777 interviews were successfully achieved with consenting antenatal care clients – 427 in intervention hospitals compared to 350 in control hospitals (2.9% non-response rate).

### Measurements

The questionnaire was adapted from Health Results-Based Financing Nigeria 2017 Exit Interview questionnaire for Antenatal care Visit by World Bank, Federal Ministry of Health, and National Bureau of Statistics [25–27]. The questionnaire contained questions organized into two sections. In section 1, we asked questions on the socio-demographic characteristics of the women – age, marital status, educational background, religion, occupation, and their number of children. In section 2, we solicited information on the ‘women’s experiences of the services provided during the antenatal visit. These questions assessed the content and experience of care during the current and previous ANC visits, which we categorized into eighteen Quality Indicators (QIs) for maternal and fetal assessment management, disease testing and management, counseling, and information sharing [28]. QIs assessed included whether or not the provider undertook the following measurements during the visit: fetal heart rate, blood pressure, palpation, height, weight, assessment of anemia, tetanus immunization, antimalarial prevention, and provision of information. Other QIs were counseling on side effects of iron pills, nutrition, delivery date, signs of complications, family planning counseling, among others. The information provided guided the data analysis and enabled us to compare the results between the intervention and control hospitals.

### Ethical approval

Ethical approval for the study was obtained from the World Health Organization and the National Health Research Ethics Committee (NHREC) of Nigeria – number NHREC/01/01/2007–16/07/2014, renewed in 2015 with NHREC 01/01/20047–12/12/2015b.

This study was registered in the ISRCTN on August 14th, 2020. Trial Registration Number ISRCTN17985403 10.1186/ISRCTN17985403 Retrospective registration. The reason for the retrospective registration is the current non-recognition of the Nigeria Clinical Trials Registry (NCTR); which is currently not an ICMJE or WHO ICTRP approved registry. (This study was registered in the Nigeria Clinical Trials Registry on April 14th, 2016. Trial Registration Number NCTR No: 91540209. http://www.nctr.nhrec.net/).

The Chief Medical Directors, Heads of Departments of the hospitals, and the participants were informed of the purpose of the study, and verbal consent was obtained from them to conduct the study. They were assured of the confidentiality of the information obtained. No names or specific contact information were obtained from study participants.

### Statistical analysis

A primary outcome of the intervention was to improve the quality of antenatal care. Hence, the analysis compared the reported eighteen quality indicators (QIs) in intervention hospitals with those in control hospitals. We summarized the differences in the characteristics of respondents in intervention and control hospitals using mean and standard deviation, absolute numbers and percentages. We grouped the QIs into three categories, maternal and fetal measures, disease testing and management, and counseling and information sharing. The response option for each QI was yes (coded 1) when it occurred and no (coded 0) when the event did not occur. All the responses were aggregated to obtain the total sum for the 18 QIs, and the sum for the three sub-categories. We assessed the QIs in two ways, as the count of reported QIs and each QI separately. The generalized linear model (GLM), Poisson regression was used to assess whether the counts of QIs at intervention hospitals were significantly higher than at control hospitals. We fit four models, the count for all QIs, and the three categories. GLM estimates were converted to odds ratios. Logistic regression was used to model the odds of reporting each QI at intervention hospitals. The Firth correction in logistic regression was used to adjust for small counts in some cells. All models were adjusted for socio-demographic characteristics and the number of visits. Alpha was set at 0.05, and all *p* values were two-sided. SAS version 9.4 and JMP 14 Pro were used for the analyses.

## Results

### Profile of the respondents

Over the 21-month period, a total of 112,347 antenatal care attendees was recorded comprising 63,012 (56%) in the intervention hospitals and 49,335 (44%) in the control sites. Data were obtained from 777 women who used the antenatal clinics at the study hospitals. Fifty-five percent (427) used intervention hospitals, while 45% (350) used control hospitals. The differences between the intervention and control hospitals are presented in Table 1. The mean age of the respondents was approximately 29 years at the intervention sites and 30 years at the control sites; the mean number of children per woman was 1.8 in both sites. The average number of attending health workers was 3.6 at the intervention sites and 4.5 at the control sites. On average, women at the intervention sites had attended four antenatal visits, compared to three visits in the control sites. Most respondents in both sites were of non-Catholic Christian religion, but there were more Muslims in the intervention sites compared to the control sites. Most of the women in the two sites attained post-secondary education; were married in a monogamous union; and were self-employed. About 26% of the respondents in the intervention sites and 27% at the control sites were primigravid. Close to one-fifth of the respondents at the intervention sites were on their first antenatal care visit compared to 28% at the control sites.

**Table 1:** Characteristics of women who attended antenatal clinics at intervention and control hospitals

	Intervention (*n* = 427)	Control (*n* = 350)
Characteristic	Mean (Sd)	Mean (Sd)
Age	28.9 (5.8)	30.0 (4.6)
Number of Children	1.8 (1.7)	1.8 (1.5)
Number of attending health workers	3.6 (1.5)	4.5 (1.1)
Number of total antenatal visits	3.6 (2.1)	2.9 (1.5)
	n (%)	n (%)
Religion		
Catholic	46 (10.9)	62 (17.7)
Other Christian denominations	232 (54.7)	238 (68.0)
Islam	146 (34.4)	50 (14.3)
Education Level		
No Education	42 (9.8)	0 (0.0)
Primary	63 (14.8)	29. (8.3)
Secondary	150 (35.1)	155 (44.3)
Higher	172 (40.3)	166 (47.4)
Age group		
15–20	37 (8.7)	10 (2.9)
21–25	85 (20.0)	55 (15.7)
26–30	144 (33.7)	114 (32.6)
31–35	103 (24.1)	133 (38.0)
36+	58 (13.6)	38 (10.9)
Marital Status		
Single/Never Married/ Co habiting (Living together)	10 (2.3)	25 (7.1)
Married (Polygamous)	68 (5.9)	14 (4.0)
Married (Monogamous)	349 (81.7)	311 (88.9)
Employment		
Not Working	124 (29.0)	68 (19.4)
Civil Servant	71 (16.6)	41 (11.7)
Self-Employed	195 (45.7)	209 (59.7)
Private Sector Employee	37 (8.7)	32 (9.1)
Read complete sentence (Yes)	130 (30.4)	70 (20.0)
First Pregnancy (Yes)	110 (25.8)	93 (26.6)
First visit to facility (Yes)	82 (19.2)	99 (28.3)

### Antenatal care quality indicators reported by women between sites

Table 2 shows the adjusted estimates for the generalized linear model, poison regression. GLM coefficients and estimates were converted to odds ratios and corresponding confidence intervals. The estimates on the far left of the table represent the model for the count of all QIs, followed by the model for the count for maternal and fetal management, disease testing and management and counseling, and information sharing on the far right. There was 11% odds of reporting higher count of all QIs (OR 1.11, 95% CI 1.06–1.17), 11% odds of reporting higher counts of disease testing and management QIs (OR 1.11, 95% CI 1.00–1.23), and 32% odds of reporting higher counts of counseling and information sharing QIs (OR 1.32, 95% CI 1.23 to 1.41) at intervention hospitals compared to control hospitals. On the contrary, there was 17% odds of reporting lower count of maternal and fetal management QIs at the intervention hospitals compared to the control sites. The factors associated with higher counts of reported QIs include an increasing number of attending health workers, first visit, and the total number of visits to the facility, education, and religious affiliation.

**Table 2:** Factors associated with the number of reported quality indicators by women who attended antenatal clinic at intervention and control hospitals in Nigeria between October 2017 and June 2019

Category	Sum of all QIs				Sum of maternal and fetal management QIs				Sum of disease testing and management QIs				Sum of counseling and information sharing QIs			
	Odds Ratio	95% CI		*P*-value	Odds Ratio	95% CI		*P*-value	Odds Ratio	95% CI		*P*-value	Odds Ratio	95% CI		*P*-value
Intervention vs control	**1.11**	**1.06**	**1.17**	**<.0001**	**0.83**	**0.77**	**0.90**	**<.0001**	**1.11**	**1.00**	**1.23**	**0.051**	**1.32**	**1.23**	**1.41**	**<.0001**
Number of attending	**1.05**	**1.04**	**1.07**	**<.0001**	**1.05**	**1.02**	**1.08**	**0.001**	0.98	0.95	1.02	0.415	**1.10**	**1.07**	**1.13**	**<.0001**
First pregnancy (Yes)	1.03	0.97	1.09	0.299	0.99	0.91	1.08	0.876	1.01	0.90	1.13	0.900	1.06	0.98	1.15	0.116
First visit to facility (Yes)	**1.14**	**1.07**	**1.20**	**<.0001**	**1.36**	**1.24**	**1.49**	**<.0001**	**1.25**	**1.10**	**1.41**	**0.0004**	**0.91**	**0.83**	**0.99**	**0.031**
Number of visits to facility	**1.02**	**1.00**	**1.03**	**0.021**	1.00	0.98	1.02	0.877	1.01	0.98	1.04	0.6325	**1.03**	**1.01**	**1.04**	**0.008**
Age group (Ref = 36+)																
15–20	0.96	0.85	1.08	0.477	1.05	0.87	1.27	0.599	1.07	0.83	1.37	0.604	0.86	0.72	1.03	0.096
21–25	1.00	0.92	1.09	0.923	1.04	0.9	1.19	0.601	1.02	0.85	1.23	0.802	0.99	0.87	1.11	0.825
26–30	1.02	0.95	1.09	0.652	1.03	0.91	1.15	0.676	1.01	0.87	1.18	0.902	1.03	0.93	1.14	0.629
31–35	0.98	0.92	1.06	0.675	1.03	0.91	1.16	0.640	1.02	0.87	1.19	0.841	0.95	0.86	1.06	0.362
Education level (Ref = College+)																
No Education	**0.82**	**0.74**	**0.92**	**0.001**	1.04	0.88	1.23	0.68	1.00	0.80	1.25	1.00	**0.65**	**0.55**	**0.78**	**<.0001**
Primary	1.00	0.94	1.08	0.91	0.99	0.88	1.11	0.87	1.07	0.92	1.25	0.38	0.98	0.89	1.09	0.726
Secondary	0.98	0.93	1.02	0.336	0.99	0.92	1.07	0.858	1.01	0.91	1.12	0.807	0.95	0.89	1.02	0.129
Religion (Ref-Islam)																
Catholic	1.06	0.98	1.14	0.122	**0.88**	**0.78**	**0.99**	**0.032**	1.14	0.97	1.34	0.117	**1.13**	**1.02**	**1.26**	**0.020**
Other Christian denominations	1.01	0.96	1.07	0.681	**0.88**	**0.80**	**0.96**	**0.005**	1.10	0.97	1.25	0.132	1.06	0.97	1.15	0.175

**Fig. 1:**
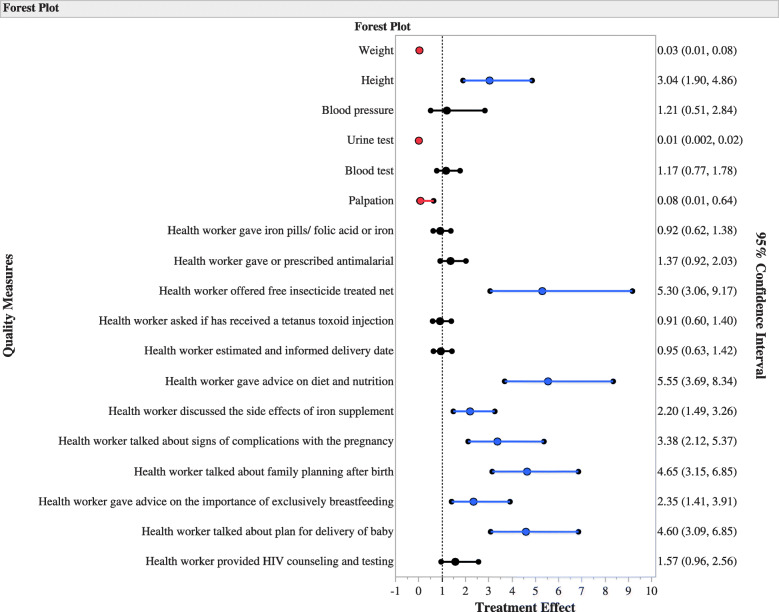
The adjusted odds of reported quality indidcators by women who attended antenatal clinic at intervention and control hospitals in Nigeria between October 2017 and June 2019

Figure 1 shows the forest plot of the odds of reporting each QI at intervention hospitals compared to the control hospitals. The intervention hospitals performed better on counseling and information sharing QIs, such as giving advice on diet and nutrition, discussing the effect of iron supplement, talking about potential complications and delivery plans, providing advice on breastfeeding and family planning. These QIs were more than twice likely to be reported at intervention hospitals. The odds of reporting that a health worker offered free insecticide-treated net (a QI for disease testing and management) were higher at the intervention hospitals compared to the control sites. On the contrary, three of the six maternal and fetal management QIs (weight measurement, urine testing, and palpation) were less likely to be reported at intervention hospitals.

## Discussion

The study was designed to investigate the effectiveness of a set of multiple interventions in improving the quality of antenatal care in ‘Nigeria’s referral hospitals. The interventions offered consisted of strategic plan development and consensus building, staff re-training, implementation of a computerized appointment system, provision of composite health education for staff and patients, maternal death audit, and advocacy for adherence to guidelines for maternal care to policymakers and hospital administrators. The quality of antenatal care was assessed by determining the extent to which providers in intervention hospitals offered 18 signal functions recommended by the World Health Organization as compared to providers in control hospitals. The signal functions were divided into three categories – maternal and fetal measures, disease testing and management, and counseling and information sharing.

The results showed that the interventions were effective in improving the quality of counseling and information sharing, and disease testing and management but less effective in maternal and fetal measures. Specifically, the results showed that there was no significant difference between intervention and control hospitals in the extent to which health providers regularly measured the blood pressure of pregnant women, provided blood tests, folic acid, and iron supplements. Also, in the provision of tetanus toxoid immunization, informed women of their estimated delivery date and counseled on HIV. These signal functions may be generic or routine in referral hospitals, and therefore the intervention had limited effects in changing the regular practices.

Of interest was that the intervention hospitals were significantly less likely than the control hospitals to measure the weights, carry out urine tests, and palpate the abdomen of the pregnant women. These three functions are likely to be modulated by the providers’ workload [29]. Therefore, the results obtained may be because the control sites had more providers (mean of 4.5) to offer these services as compared to intervention hospitals that had a mean of 3.6 providers per clinic days. By contrast, the results show that intervention hospitals were significantly more likely to measure the height of pregnant women, offer insecticide-treated bed nets, counsel women on diet and nutrition, provide information on the complications of pregnancy. Also, they were more likely to counsel the women on family planning after delivery, exclusive breastfeeding, and outline plans to the women for the delivery of the baby. These beneficial effects of the intervention on these QIs may be due to the features of the intervention that focused mainly on health education, staff training, and information sharing.

The pattern of the results indicates that it is likely that the training component of the intervention focused mainly on staff-client interactions rather than the technical aspects of the provision of care in the antenatal clinics. Furthermore, since the intervention did not change the supply side of antenatal care provision in the hospitals, it is possible that lack of clinical materials in the intervention hospitals may have hindered the ability of providers to offer clinical care that is dependent on the availability of clinical supplies.

Bruce (1990) proposed seven domains for assessing the quality of care to include:” choice of methods, the information given to patients, technical competence, interpersonal relations, follow-up and continuity mechanisms, and the appropriate constellation of services” [30]. With this analysis in mind, it is evident that our intervention may have focused more on the information given to patients, interpersonal relations, and technical competence, and less on addressing the appropriate constellation of services and choice of methods. Although the intervention included an advocacy component to policymakers and health administrators to promote adequate allocation of resources and increased budget for service delivery to the hospitals, this did not appear to have yielded any positive results during the period. For low resource settings, the allocation of resources to address facility deficit is often painstakingly slow. As shown by the results of this study, interventions designed to solicit third party support for supply improvements may not likely succeed. Going forward, we recommend that interventions that seek to address the quality of antenatal care in low-resource settings should also focus on improving the supply side to ensure that all elements of care are engrained in the intervention and implementation processes.

The study had both limitations and strengths. One of the limitations of this study is the quasi-experimental design. The respondents were not randomly assigned to the experiment and control groups; thus, selection bias could not have been totally eliminated as well as other threats to validity such as history, and testing. Other limitations include the fact that only four hospitals – two intervention and two control hospitals – were involved in the research process. A larger number of hospitals, including private and non-profit hospitals, would have allowed a better assessment of the hospital types as determinants of quality of antenatal care. A larger number of hospitals would have also allowed a larger sample size, and therefore, a more robust assessment of the intervention. However, we were limited by resource constraints. Also, we aimed to have a manageable sample size that would allow a deeper implementation of the activities and a more accurate measurement of the indicators.

The study is also limited by the fact that it was conducted in two out of the six geo-political zones of the country, which may reduce the external validity of the results. However, the four hospitals are large and standard referral public hospitals in the country that offer services to wide catchment areas that cover adjoining states and geo-political zones. As shown, 63,012 women used the intervention hospitals for antenatal clinics during the period compared to 49.335 women that used the control hospitals, which further testify to the large volume of patients attended to by the intervention hospitals. Hence, the results can be generalized to referral public health facilities in the country.

A major concern when a complex intervention comprising multiple activities is implemented is the need to isolate the specific component of the intervention that accounts for the success or non-success of the intervention [31, 32]**.** We are unable to do this for this study, but based on the results obtained, we believe that the health education component and the staff training may have been most impactful. However, the strategic plan development where the hospital staff was able to reach a consensus on the need to reduce the high rate of maternal mortality through better implementation of antenatal care may also have had positive effects.

A major strength of the study is the fact that the intervention activities were identified from the results of formative research that investigated the determinants of quality of antenatal care in the hospitals [18, 19]. This was followed by the co-design of the interventions by the research teams in collaboration with the hospitals’ administrators and health providers. This process has not only helped the effective implementation of the activities but will ensure the sustainable integration of the project milestones and processes into the service delivery structure of the intervention hospitals. By sharing the results with officials in the control hospitals, we hope the control hospitals and similar hospitals in the country will adopt some components of the intervention to improve their quality of antenatal services. Another strength of this study is the use of exit interviews, which allowed the prospective and early recall of events that took place during the antenatal clinic visits in contrast to retrospective population-based studies previously conducted in Nigeria [15, 33]. This increased the accuracy of recall of the events, and thereby the internal validity of the results. The interviews were conducted by trained staff who were not part of the research process, which potentially enhanced the accuracy and validity of the results.

In conclusion, the results of this study indicate that a complex intervention identified by stakeholders that comprise strategic plan development, staff training, computerized referral system, and health information was effective in improving the quality of counseling and information sharing, disease testing and management during antenatal care in ‘Nigeria’s referral hospitals. However, the intervention was less effective in improving the quality of maternal and fetal care. We recommend that interventions that address the quality of antenatal care should include all elements of care, including the quality supply of clinical facilities as well as resource mobilization.

## Data Availability

The dataset used during this study are available from the corresponding author on reasonable request.

## Abbreviations

WHO: World Health Organization
NDHS: Nigerian Demographic and Health Survey
BCC: Behavioral Change Communication
MDRS: maternal death reviews and surveillance
QIs: Quality Indicators
GLM: generalized linear model
